# Comparison of Local Control and Toxicity in T4 Nasopharyngeal Carcinoma Patients Treated with Induction Chemotherapy and Intensity-Modulated Radiotherapy: Conventional vs. Hyperfractionated Regimens

**DOI:** 10.3390/medicina62010076

**Published:** 2025-12-30

**Authors:** He-Yuan Hsieh, Jin-Ching Lin, Hen-Hong Chang

**Affiliations:** 1 Graduate Institute of Integrated Medicine, College of Chinese Medicine, and Chinese Medicine Research Center, China Medical University, Taichung 404, Taiwan; 2 Department of Radiation Oncology, Taichung Veterans General Hospital, Taichung 407, Taiwan; 3 Institute of Clinical Medicine, School of Medicine, National Yang-Ming Chiao-Tung University, Taipei 112, Taiwan; 4 Department of Post-Baccalaureate Medicine, College of Medicine, National Chung Hsing University, Taichung 402, Taiwan; 5 Department of Radiation Oncology, Changhua Christian Hospital, Changhua 500, Taiwan; 6 Department of Chinese Medicine, China Medical University Hospital, Taichung 404, Taiwan

**Keywords:** T4 nasopharyngeal carcinoma, hyperfractionated, intensity-modulated radiotherapy, induction chemotherapy

## Abstract

*Background and Objectives*: T4 nasopharyngeal carcinoma (NPC) is a locally advanced disease with a high risk of local recurrence despite advances in radiotherapy techniques. Induction chemotherapy followed by intensity-modulated radiotherapy (IMRT) is a standard approach, but the optimal fractionation strategy remains uncertain. We conducted a retrospective study comparing survival and local control rates in T4 NPC patients treated with induction chemotherapy followed by IMRT using either conventional fractionation (CF) or hyperfractionation (HF). *Methods*: This study included 171 non-metastatic T4 NPC patients treated with induction chemotherapy and IMRT/arc therapy (volumetric-modulated arc therapy and TOMOTHERAPY) between 2003 and 2021. The patients were divided into two groups: the CF group (109 patients) received 70 Gy in 35 fractions, while the HF group (62 patients) received 76.8 Gy in 64 fractions (1.2 Gy twice daily). The most commonly used induction chemotherapy regimen was weekly cisplatin and fluorouracil/leucovorin. The primary endpoint was the local control rate between the two treatment groups. Secondary endpoints were adverse events, overall survival, regional recurrence, and distant metastasis rates. Competing risk analyses based on the subdistribution hazard model and cumulative incidences were calculated. *Results*: Over a median follow-up of 74 months, the 5-year local control rate was higher in the CF group than in the HF group (83.6% vs. 74.7%; *p* = 0.0336). Two prognostic factors were significantly associated with local control, namely hyperfractionated RT (subdistribution hazard ratio [SHR] 2.259, 95% confidence interval [CI] 1.159–4.403, *p* = 0.0167) and arc therapy (SHR 0.267, 95% CI 0.127–0.565, *p* = 0.0006). Acute toxicities (≥grade 3 dermatitis and mucositis) were more frequent in the HF group (16.1% vs. 11.9%; 22.6% vs. 11%), as were late adverse events (33.9% vs. 22%), particularly hearing loss. No significant differences were noted in 5-year regional control (99.0% vs. 96.3%; *p* = 0.0522), distant metastasis (84.7% vs. 78.0%; *p* = 0.3307), or overall survival (71.2% vs. 64.2%; *p* = 0.4704). *Conclusions*: Induction chemotherapy followed by CF-IMRT offers better local control and fewer adverse events than HF-IMRT in T4 NPC patients, making CF-IMRT the preferred treatment.

## 1. Introduction

Nasopharyngeal carcinoma (NPC) is a malignancy endemic to East and Southeast Asia [1]. Despite advancements in treatment, local recurrence remains a significant challenge, particularly for patients with advanced T-stage disease. The local recurrence rate after definitive radiotherapy (RT) ranges from 10% to 20%, with higher rates observed in patients initially diagnosed at an advanced T stage [2]. Achieving satisfactory local control for advanced T-stage NPC is difficult due to the tumor’s proximity to critical normal structures such as the brainstem, spinal cord, and optic chiasm. While modern radiation techniques have improved locoregional control, factors such as skull base invasion, intracranial extension, and tight radiation field margins near the clivus continue to predict poorer local control [3].

Induction chemotherapy has emerged as a highly effective strategy in the management of locoregionally advanced NPC. Regimens such as gemcitabine/cisplatin and cisplatin/fluorouracil/docetaxel, when administered before concurrent chemoradiotherapy, have been shown to significantly improve treatment outcomes [4,5]. Moreover, weekly administration of cisplatin and fluorouracil/leucovorin has also demonstrated particularly promising outcomes—not only favorable 5-year overall and progression-free survival rates, but also high complete response rates, low relapse rates, significantly reduced ≥ grade III hematologic toxicities, and minimal interruptions to treatment [6,7].

Importantly, to mitigate the treatment-related toxicities associated with concurrent chemoradiotherapy, growing evidence supports the use of induction chemotherapy followed by RT alone as an alternative approach [8,9,10,11,12]. To date, induction chemotherapy followed by RT alone is gaining recognition as a viable and less toxic treatment paradigm for patients with non-metastatic T3–4N1–3 or T1–4N2–3 NPC [13].

In addition, hyperfractionated RT has been investigated as a potential approach to improve local control in NPC patients, but its benefits remain under debate. Early studies utilizing accelerated hyperfractionated RT with two-dimensional (2D) techniques reported significant neurological complications [14]. These complications may be attributed to the higher biologically effective doses (84.4 Gy) and the less precise targeting of the primary tumor. However, some studies have demonstrated that hyperfractionated RT can improve outcomes in certain settings [15,16]. Moreover, concurrent chemoradiotherapy using three-dimensional conformal RT (3D-CRT) and hyperfractionation (1.2 Gy per fraction, two fractions per day, Monday to Friday, for a total dose of 74.4 Gy) has been demonstrated to provide better local control and survival rates for patients with T3 and T4 lesions [16].

For T4 NPC patients, the clinical challenge lies in achieving adequate coverage of the upward-invasive primary tumor while protecting adjacent intracranial critical structures. Induction chemotherapy provides a valuable solution by inducing significant tumor shrinkage before RT. Moreover, the use of precise RT techniques, such as intensity-modulated radiotherapy (IMRT), is strongly recommended by the National Comprehensive Cancer Network (NCCN) guidelines as the preferred approach for patients with NPC to minimize radiation exposure to critical structures [13]. Additionally, reducing the target volumes of IMRT after induction chemotherapy in patients with locoregionally advanced NPC does not increase the risk of locoregional relapse, while potentially improving quality of life and reducing late-treatment-related toxicity [17]. Despite these advancements, limited data are available on the outcomes of combining induction chemotherapy with hyperfractionated or conventional fractionated IMRT specifically for T4 NPC patients.

To address this gap, we conducted a retrospective study to evaluate survival outcomes, with a particular focus on local control rates, in T4 NPC patients who underwent induction chemotherapy followed by IMRT with either conventional fractionation (CF group) or hyperfractionation (HF group). This analysis aims to provide insights into optimizing treatment strategies for this challenging patient population.

## 2. Materials and Methods

### 2.1. Patients, Study Design, and Treatment

This retrospective study was approved by the institutional review boards of our institution (Taichung Veterans General Hospital, TCVGH-IRB No. CE25008B). The inclusion criteria were as follows: (1) previously untreated, biopsy-proved NPC; (2) clinical stage T4 disease, as determined by the 7th edition of the American Joint Committee on Cancer staging manual; (3) Karnofsky performance score (KPS) > 60; (4) received induction chemotherapy followed by RT or concurrent chemoradiotherapy; (5) RT with IMRT technique or arc therapy (volumetric-modulated arc therapy and TOMOTHERAPY); and (6) follow-up duration of more than 12 months. Patients were excluded from the study if they met any of the following criteria: (1) pregnancy or breastfeeding; (2) distant metastases detected at the initial diagnosis; (3) definite concurrent chemoradiotherapy; (4) RT administered using older techniques, such as 2D RT or 3D-CRT; or (5) incomplete or insufficient medical records. All patients underwent comprehensive initial staging, which included a physical examination, detailed medical history, and baseline laboratory testing. Laboratory assessments encompassed blood count and serologic biochemistry, including liver and renal function tests. Imaging studies included chest radiography, magnetic resonance imaging of the head and neck, abdominal ultrasonography, and whole-body bone scans. Additionally, 18F-fluorodeoxyglucose positron emission tomography (18F-FDG PET) scans were performed at the discretion of the attending physician.

**Figure 1:**
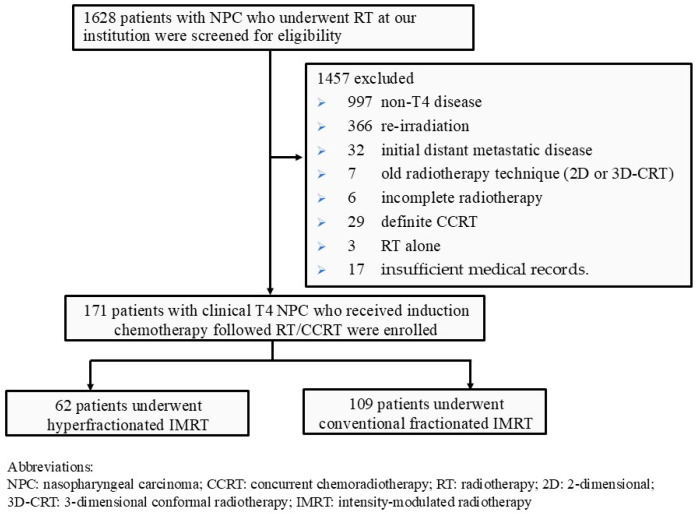
The flow chart of patient enrollment.

Between June 2003 and December 2021, a total of 171 patients were enrolled in our study evaluating treatment outcomes for cancer therapy. The detailed inclusion/exclusion flow chart is displayed in Figure 1.

All patients underwent induction chemotherapy, followed by either RT alone (168 patients) or concurrent chemoradiotherapy (3 patients and all in the CF group). Among these patients, 109 were in the CF group and 62 were in the HF group. Radiotherapy was administered using 6 MV photon beams, with treatment protocols tailored to the specific type of fractionation employed. The gross tumor volume of nasopharynx (GTVp) and neck lymph nodes (GTVn) was delineated according to the contrast-enhanced computed tomography simulation after induction chemotherapy. The bony destructions were included in the GTVp according to pre-treatment magnetic resonance imaging. The clinical target volume 1 (CTV1) was generated by adding a 5–10 mm margin to the GTVp and GTVn and regions with a high risk of microscopic invasion, including posterior maxillary sinus, ethmoid sinus, sphenoid sinus, cavernous sinus, bilateral foramen ovale, foramen rotundum, foramen lacerum, posterior nasal cavity, pterygoid fossa, pterygopalatine fossa, whole parapharyngeal space, basilar clivus, and high-risk cervical lymphatic regions according to pre-treatment image. The CTV2 was defined as the low-risk neck lymphatic regions. The planning target volume (PTVp, PTVn, PTV1, and PTV2) was obtained via the expansion of 3–5 mm margins around the corresponding CTV to compensate the uncertainties. For the CF group, the prescribed doses were 70 Gy in 35 fractions to PTVp and PTVn (ranging from 66 to 76 Gy in 33 to 38 fractions; 2 Gy per fraction, once daily, 5 fractions per week); 60–66 Gy in 35 fractions to PTV1; and 50–54 Gy in 35 fractions to PTV2. For the HF group, the prescribed doses were 76.8 Gy in 64 fractions to PTVp and PTVn (ranging from 72 to 78.6 Gy in 60 to 64 fractions; 1.2 Gy per fraction, twice daily with interval > 6 h, Monday through Friday), 66–70.4 Gy in 64 fractions to PTV1, and 50–56 Gy in 64 fractions to PTV2. The quality of the plan was evaluated according to the homogeneity index (HI) and conformity index (CI). The HI was calculated using (D_2%_ − D_98%_)/D_50%_. The CI was defined as CI = C1 × C2, and C1 = V_CTVref_/V_CTV_, C2 = V_CTVref_/V_ref_, where V_CTVref_ is CTV receiving the reference dose or above, V_CTV_ is the volume of CTV contouring, and V_ref_ is the body volume receiving the reference dose or above. The dosimetric date was presented at Supplement Table S1. The mean CI for both the CF group and HF group was 0.84. The mean HIs for the CF group and HF group were 0.10 and 0.12, respectively (the detailed data are shown in Supplementary Table S1).

The most common induction chemotherapy regimen, administered to 155 patients (90.6%), was the weekly cisplatin–fluorouracil/leucovorin regimen, consisting of cisplatin 60 mg/m^2^ on day 1 and fluorouracil 2500 mg/m^2^ plus leucovorin 250 mg/m^2^ intravenous infusion for 24 h on day 8, which was repeated every 2 weeks for 5–6 cycles. Among the patients, 147 (94.8%) could tolerate this regimen for more than 5 cycles. Six patients in the CF group and one patient in the HF group received the induction chemotherapy for <5 cycles. In total, 78 patients received IMRT, 66 volumetric-modulated arc therapy, and 27 TOMOTHERAPY. Three patients in the CF group received concurrent chemotherapy with weekly cisplatin 30–40mg/m^2^. The response after induction chemotherapy was classified into complete response, partial response, and stable disease according to response evaluation criteria in solid tumors (RECIST) version 1.1 [18].

### 2.2. Follow-Up, Endpoints, and Statistical Analysis

Post-treatment follow-up was scheduled every month for 3–6 months, followed by every 3 months for 3 years and every 4–6 months thereafter. Examinations included physical examinations, routine complete blood cell counts, serologic biochemistry data, and fiberscopic examinations at regular follow-up. Head and neck computed tomography imaging was scheduled every 3 months for 3 years and every 6 months for 4–5 years and then annually thereafter. Chest radiography, abdominal sonography, and whole-body bone scans were scheduled every 6 months for 3 years and then annually thereafter.

The primary endpoint of the study was to compare the local control rate between the CF group and HF group. Secondary endpoints included the assessment of treatment-related acute and late adverse events (AEs), overall survival, regional control rate, and distant metastasis-free survival rate for the two RT approaches. Acute and late toxicities were recorded based on the National Cancer Institute Common Terminology Criteria for Adverse Events (CTCAE) version 3.0 [19]. Competing risk analysis based on the subdistribution hazard model with death as a competing event was used to identify prognostic factors. The prognostic factors included RT frequency (CF vs. HF), age (≥60 vs. <60 years old), sex (men vs. women), N stage (N2-3 vs. N0-1), histology (keratinizing vs. nonkeratinizing), and radiation technique (IMRT vs. arc therapy). The cumulative incidence function of local control was conducted by Gray K-Sample test. Overall survival was defined as the start day of induction chemotherapy to the date of death of any cause or the date of the last follow-up. Survival curves were compared using the Kaplan–Meier method, and a log-rank test was used to compare the differences between the two treatment groups. Patient characteristics, treatment-related acute and late AEs, and survival outcomes were compared between the two groups using Fisher’s exact test. All statistical analyses were conducted using SAS version 9.4, with a 2-sided *p* < 0.05 considered statistically significant.

## 3. Results

### 3.1. Patients’ Characteristics

This study enrolled 126 men and 45 women. Among them, 59 and 112 patients had clinical N0-1 and N2-3 disease, respectively. Based on histological classification, 167 were classified with nonkeratinizing carcinoma and 4 with keratinizing carcinoma. A higher prevalence of N2-3 diseases (65.5%) was found for T4 NPC patients. The complete and partial response rates after induction chemotherapy were 73% and 24%, respectively. There were no significant differences between the CF group and HF group regarding age, sex, clinical N stage, KPS, tumor response to induction chemotherapy, or RT techniques. Baseline characteristics for the CF and HF groups are summarized in Table 1.

### 3.2. Local Control Rate

**Figure 2:**
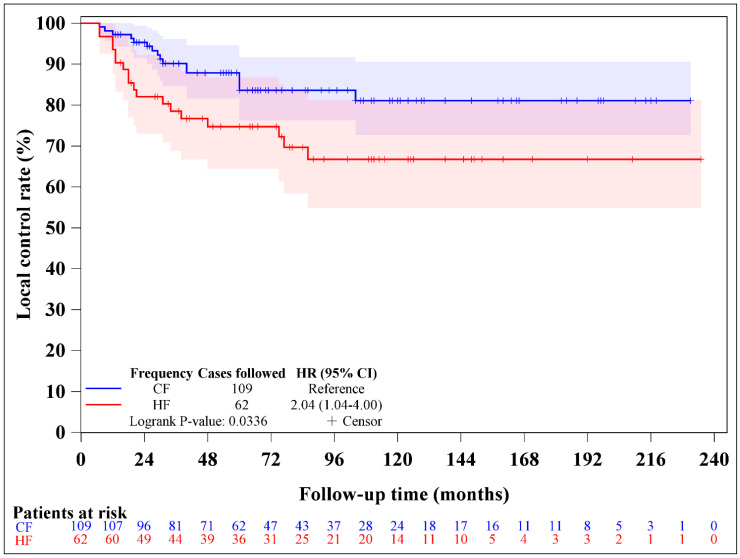
The local control rates between conventional fractionation (CF) and hyperfractionation (HF) intensity-modulated radiotherapy following induction chemotherapy for patients with T4 nasopharyngeal carcinoma (*p* = 0.0336).

**Figure 3:**
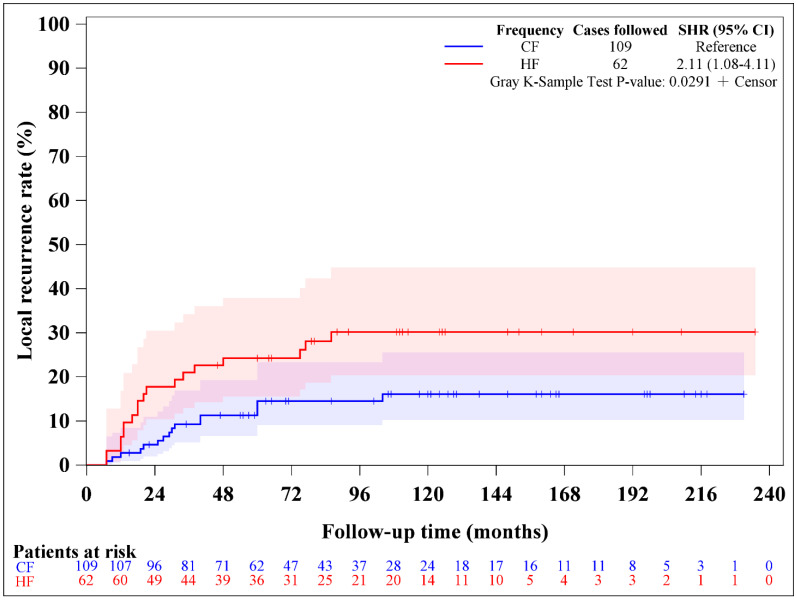
Cumulative incidence function of local recurrence rate between conventional fractionated (CF) and hyperfractionated (HF) intensity-modulated radiotherapy in patients with T4 nasopharyngeal carcinoma who received induction chemotherapy (*p* = 0.0291).

Local recurrence was observed in 16 patients in the CF group and 18 patients in the HF group. The median durations of local recurrence for the entire cohort, the CF group, and the HF group were 67 months (interquartile range [IQR] 33–111 months), 66 months (IQR 35–110 months), and 71 months (IQR 31–113 months), respectively. The 5-year local control rates were 83.6% for the CF group and 74.7% for the HF group (log-rank *p* = 0.0336, Figure 2). The competing risk analyses for local control are summarized in Table 2. The univariate analyses identified two prognostic factors significantly associated with local control, including hyperfractionated RT (subdistribution hazard ratio [SHR] 2.108, 95% confidence interval [CI] 1.080–4.111, *p* = 0.0288) and arc therapy (SHR 0.280, 95% CI 0.131–0.595, *p* = 0.0009). Both prognostic factors remained significant in the multivariate analysis. The competing risk analysis using the cumulative incidence function also confirmed this consistent result (Figure 3). Subgroup analysis showed no difference in local control between HF and CF groups when using the IMRT technique [SHR 1.681, 95% CI 0.769–3.672, *p* = 0.1930] but significantly poor control in the HF group when using arc therapy [SHR 5.829, 95% CI 1.220–27.835, *p* = 0.0271].

For salvage treatment, 14 patients in the CF group underwent re-irradiation. Of these, eight received a curative dose of 51–70 Gy, while six received a palliative dose of 8–32 Gy. In the HF group, nine patients underwent re-irradiation. Among them, two received 60 Gy in 20–25 fractions, four received a hypofractionated dose of 18–50 Gy in 2–5 fractions, and three received a palliative dose of 12–45 Gy in 5–15 fractions.

### 3.3. Treatment-Related Acute and Late AEs and Survival Outcomes

#### 3.3.1. Acute and Late AEs

The details of acute and late AEs are shown in Table 3. Patients in the HF group had a higher proportion of ≥grade 3 acute radiation dermatitis (16.1% vs. 11.9%) and mucositis (22.6% vs. 11%,) compared to the CF group. Late-treatment-related AEs were also more common in the HF group (33.9% vs. 22.0%). The most frequently reported late AEs included hearing loss (*n* = 35; there were 7, 23, and 5 patients with grade 1, 2, and 3 hearing loss, respectively), dysphagia (*n* = 8; there were 2, 4, and 2, patients with grade 1, 2, and 3 dysphagia, respectively), carotid artery stenosis/occlusion/stroke (*n* = 7), and skull base osteoradionecrosis (*n* = 2).

#### 3.3.2. Overall Survival, Regional Recurrence, and Distant Metastasis

After a median follow-up of 74 months (IQR 44–125 months), the 3-year and 5-year overall survival rates were 81.4% vs. 83.9% and 71.2% vs. 64.2% for the CF and HF groups, respectively (log-rank *p* = 0.4704).

Regional recurrence was observed in four patients in the HF group and one patient in the CF group. The median time of regional recurrence for the entire cohort, the CF group, and the HF group was 74 months (IQR 44–125 months), 70 months (IQR 44–124), and 75 (IQR 46–125). The 5-year regional control rates for the CF group and HF group were 99.0% and 96.3%, respectively (log-rank *p* = 0.0522). One patient experienced retropharyngeal lymph node recurrence and underwent salvage chemotherapy with a local RT boost. Another patient developed upper neck lymph node metastasis and was treated with neck lymph node dissection followed by adjuvant chemotherapy. Two patients had simultaneous local and regional recurrences; one underwent nasopharyngectomy and neck lymph node dissection followed by adjuvant chemotherapy, while the other was enrolled in a clinical trial. Additionally, one patient experienced both regional recurrence and distant metastasis and received palliative chemotherapy.

Distant metastasis occurred in 13 patients in the HF group and 16 patients in the CF group. The median times of distant metastasis for the entire cohort, the CF group, and the HF group were 67 months (IQR 35–121 months), 67 months (IQR 37–120), and 71 months (IQR 35–125), respectively. The 5-year distant metastasis-free survival rates were 84.7% in the CF group and 78.0% in the HF group (log-rank *p* = 0.3307). Patients who developed distant metastatic disease would receive palliative chemotherapy with or without palliative RT.

The competing risk analyses for regional recurrence or distant metastasis did not find any prognostic predictor.

## 4. Discussion

Hyperfractionated 3D-CRT combined with concomitant chemotherapy can improve local control and survival rates for patients with T3-4 NPC [16]. Induction chemotherapies have also been shown to be an effective therapeutic strategy for patients with locally advanced nasopharyngeal carcinoma [4,5]. However, there is limited evidence regarding the outcomes of combining induction chemotherapy with hyperfractionated or conventional fractionated IMRT specifically for patients with T4 NPC. To our knowledge, this is the first study to compare the treatment outcomes between different fractionated IMRT (CF group vs. HF group) in T4 NPC patients who have received induction chemotherapy. Our findings reveal that induction chemotherapy followed by conventional fractionated IMRT provides better local control than hyperfractionated IMRT, without significant differences in regional control, distant metastasis, or overall survival.

According to the study of Jian et al., hyperfractionated 3D-CRT (total dose 74.4 Gy) in combination with concomitant chemotherapy for 37 T4 NPC patients could achieve 3-year rates of locoregional control, disease-free survival, and overall survival of 91%, 62%, and 63%, respectively [16]. In the current study, 62 patients with T4 NPC received induction chemotherapy followed by hyperfractionated IMRT (median dose 76.8 Gy, range 70.8–78.6 Gy) and showed a 3-year local control rate of 78%, a regional control rate of 96%, and an overall survival rate of 81%. The smaller coverage area in the IMRT planning may be the cause of the poorer local control rate as compared with the 3D-CRT planning. However, the modern technique of IMRT provided more precise delivery of radiation dose to the target volume with sparing of adjacent normal structures, which achieved better survival outcome when compared with 3D-CRT.

Induction chemotherapies have been demonstrated to be a significant effective strategy for patients with locally advanced NPC in terms of better local control and survival outcomes [4,5]. Zhang et al. demonstrated that induction chemotherapy with a gemcitabine and cisplatin regimen followed by concomitant chemoradiotherapy significantly improved recurrence-free survival and overall survival compared to chemoradiotherapy alone. They reported that the 3-year recurrence-free survival and overall survival rates were 85.3% and 94.6%, respectively [4]. There were 242 patients who received induction chemotherapy, of whom 109 patients belonged to T4 NPC, and all of these patients had KPS ≥ 80. Our study focused on T4 NPC patients and showed a 3-year local control rate, regional control rate, and overall survival of 90%, 99%, and 85% in the CF group, respectively. Our results showed a similar local control rate but a relatively low survival outcome in comparison with Zhang’s study. This may be attributed to the older age (24.8% of patients aged ≥ 60) in the CF group than in the HF group (12.9%).

Jen et al. published a study examining dose escalation using twice-daily RT for NPC and showed that the incidence of acute AEs (mucositis and moist desquamation) was higher in the twice-daily group than in the once-daily group [20]. Although modern IMRT was applied to patients with T4 NPC, our study displayed the similar finding that patients in the HF group still had a higher incidence of ≥grade 3 acute AEs than patients in the CF group. The reported incidence of late-treatment-related AEs for stage I–IV non-metastatic NPC patients undergoing hyperfractionated RT (2D RT or 3D-CRT) included 9–11% cranial nerve palsy, 4% temporal lobe or brainstem damage, 39% otitis media, 17% trismus, and 3.4% temporal lobe necrosis [20,21,22]. In contrast, the incidence of late-treatment-related AEs for stage I–IV non-metastatic NPC patients undergoing conventional 3D-CRT included 32.0% otitis media, 39% neck fibrosis, and 14% trismus [20]. In our current study, 21 patients (33.9%) in the HF group and 24 patients (22.0%) in the CF group had late complications, including hearing loss (*n* = 35), dysphagia (*n* = 8), carotid artery stenosis/occlusion/stroke (*n* = 7), and skull base osteoradionecrosis (*n* = 2). Even though the IMRT technique was applied to T4 NPC patients, the late-treatment-related AEs were still high due to intracranial invasion disease.

Hyperfractionated RT has been extensively studied in the treatment of head and neck cancers, demonstrating significant survival benefits. A long-term follow-up and large-scale meta-analysis found that hyperfractionated RT led to absolute survival differences of 8.1% at five years and 3.9% at ten years compared to conventional fractionation [23]. These findings suggest that hyperfractionation may improve treatment outcomes in patients with head and neck malignancies. However, it is important to note that this meta-analysis excluded patients with NPC, making the efficacy of hyperfractionated RT in this specific subgroup uncertain.

NPC is a distinct type of head and neck cancer that exhibits unique biological and clinical characteristics. Standard treatment typically involves IMRT combined with chemotherapy, especially for patients with locally advanced disease. Studies by Jian et al. [16] and Jen et al. [20] suggest that hyperfractionated RT combined with concomitant chemotherapy may improve local control in T3–T4 NPC patients. However, this approach is also reported to have significantly increased treatment-related toxicities [20]. These toxicities, including severe mucositis, xerostomia, and hematologic toxicities, may compromise patient quality of life and limit the feasibility of dose escalation strategies.

In our study, we found that for T4 NPC patients who first underwent induction chemotherapy, conventional fractionation appears to be preferable to hyperfractionated RT. Induction chemotherapy is increasingly utilized in NPC treatment to reduce tumor burden before RT and improve overall survival outcomes. Our findings suggest that the benefits of hyperfractionated RT may not outweigh the associated toxicities in this subset of patients, emphasizing the importance of individualized treatment strategies based on disease stage and prior therapies.

Despite the concerns regarding toxicity, emerging evidence supports the use of hyperfractionated IMRT in specific NPC populations. Hyperfractionated IMRT has been shown to significantly reduce severe late complications and improve overall survival in patients with locally advanced recurrent NPC [24]. This suggests that advances in RT techniques may mitigate some of the adverse effects historically associated with hyperfractionation while maintaining its potential benefits. The ability of IMRT to deliver precise radiation doses with better sparing of adjacent normal tissues makes it a promising modality for optimizing hyperfractionated RT strategies in NPC.

Further research is needed to clarify the role of hyperfractionated RT in NPC, particularly in the context of evolving systemic therapies and RT technologies. Prospective randomized trials comparing hyperfractionated with conventional fractionation in different NPC subgroups would be invaluable in determining the optimal radiation approach. Additionally, studies focusing on the integration of novel systemic agents, such as immune checkpoint inhibitors, with hyperfractionated RT may provide new insights into improving outcomes while minimizing treatment-related toxicities.

Although hyperfractionated RT has demonstrated clear benefits in head and neck cancers, its role in NPC remains an area of ongoing investigation. The decision to use hyperfractionation should consider tumor stage, prior treatments, and the risk of increased toxicity. Advancements in IMRT and personalized treatment strategies may further refine the application of hyperfractionated RT in NPC, ultimately improving patient outcomes while minimizing adverse effects.

This study has several limitations, including its retrospective design, small sample size, difference in patient numbers between the CF (109 patients) and HF (62 patients) groups, a lack of biomarker analysis (such as plasma EBV DNA), and the use of older induction chemotherapy regimens. Nevertheless, the older regimen of induction chemotherapy with weekly cisplatin and fluorouracil/leucovorin has previously been shown to provide robust efficacy in NPC patients, demonstrating not only an excellent tumor response rate but also remarkably low toxicity. Plasma EBV DNA is an important prognostic marker in patients with NPC. However, complete data were not available in this study, as the test is not covered by standard care in our country and must be self-funded by patients. Additionally, a detailed evaluation of late-treatment-related AEs was lacking, which limits our ability to comprehensively assess long-term toxicity profiles.

However, our study remains clinically relevant for several reasons. First, to our knowledge, this is the first investigation to directly compare hyperfractionated or conventional fractionated IMRT in T4 NPC patients following induction chemotherapy. As radiation therapy remains a cornerstone in the management of locally advanced NPC, understanding the impact of different fractionation schemes on treatment outcomes and toxicity profiles is critical, particularly in T4 disease, where local control is challenging due to tumor proximity to critical structures. Second, our findings offer preliminary yet valuable insights that could inform future prospective studies and clinical trials. While hyperfractionated RT shows promise in improving outcomes for patients with recurrent NPC requiring reirradiation, it may be less suitable for de novo T4 cases following induction chemotherapy. Lastly, while systemic treatment protocols continue to evolve, optimizing the RT component remains a key part of comprehensive care. We believe our results provide a foundation for integrating modern chemotherapy regimens with refined RT approaches in future studies.

## 5. Conclusions

Induction chemotherapy followed by conventional fractionated IMRT offers significantly better local control than hyperfractionated IMRT for T4 NPC patients. However, the observed high rates of late complications highlight the need for careful planning and management of treatment-related toxicities. Future prospective studies should incorporate modern induction chemotherapy regimens and focus on optimizing the balance between treatment efficacy and toxicity. Rigorous clinical investigations will be essential to identifying the most effective treatment strategies for patients with T4 NPC, ultimately improving both survival outcomes and quality of life.

## Figures and Tables

**Table 1:** Baseline characteristics for patients with T4 nasopharyngeal carcinoma who received induction chemotherapy followed by either conventional fractionation or hyperfractionation radiotherapy.

	Conventional Fractionation		Hyperfractionation		
Variables	(*n* = 109)		(*n* = 62)		*p*-Value
	*n*	%	*n*	%	
Age, years (median/range)	50.6 (15.3–81.4)		50.7 (26.2–77.5)		0.8496
Age					
<60	82	75.2	54	87.1	0.0770
≥60	27	24.8	8	12.9	
Sex					
Women	26	23.9	19	30.7	0.3687
Men	83	76.2	43	69.4	
Histology					
Nonkeratinizing	107	98.2	60	96.8	0.6215
Keratinizing	2	1.8	2	3.2	
Clinical N stage					
N0-1	38	34.9	21	33.9	0.9999
N2-3	71	65.1	41	66.1	
Karnofsky performance status					
≥80	92	84.4	53	85.5	0.9999
<80	17	15.6	9	14.5	
Tumor response after induction chemotherapy					
Complete response	85	78.0	40	64.5	0.1519
Partial response	21	19.3	20	32.3	
Stable disease	3	2.8	2	3.2	
Techniques of radiotherapy					
IMRT	51	46.8	27	43.6	0.7502
Arc therapy	58	53.2	35	56.5	

Arc therapy included volumetric-modulated arc therapy and TOMOTHERAPY. IMRT: intensity-modulated radiotherapy.

**Table 2:** Competing risk analyses of local control using the subdistribution hazards models, treating death as a competing event for patients with T4 nasopharyngeal carcinoma who received induction chemotherapy followed by radiotherapy.

		Univariate Model			Multivariate Model		
		SHR	95% CI	*p*-Value	SHR	95% CI	*p*-Value
RT frequency	HF vs. CF	2.108	(1.080–4.111)	0.0288	2.259	(1.159–4.403)	0.0167
Techniques of RT	Arx therapy vs. IMRT	0.280	(0.131–0.595)	0.0009	0.267	(0.127–0.565)	0.0006
Age	≥60 vs. <60	1.079	(0.466–2.502)	0.8589			
Sex	Men vs. women	1.465	(0.636–3.371)	0.3694			
N stage	N2-3 vs. N0-1	1.071	(0.523–2.194)	0.8506			
KPS	<80 vs. ≥80	1.559	(0.662–3.675)	0.3097			
Histology	Keratinizing vs. nonkeratinizing	10119	(0.200–6.259)	0.8982			

SHR: subdistribution hazard ratio; CI: confidence interval; RT: radiotherapy; CF: conventional fractionation; HF: hyperfractionated; KPS: Karnofsky performance status; IMRT: intensity-modulated radiotherapy.

**Table 3:** Acute and late toxicities for patients with T4 NPC who received induction chemotherapy followed by conventional fractionated or hyperfractionated intensity-modulated radiotherapy.

Adverse Events	Conventional fractionation (*n* = 109)		Hyperfractionation (*n* = 62)		
	*n*	%	*n*	%	*p*-Value
Acute					
Mucositis					
Grade 1	60	55.05	29	46.77	0.1407
2	37	33.94	19	30.65	
3	12	11.01	14	22.58	
Dermatitis					
Grade 1	52	47.71	24	38.71	0.5407
2	44	40.37	28	45.16	
3	12	11.01	10	16.13	
4	1	0.92	0	0.00	
Late					
Hearing loss					
Grade 1	5	4.59	2	3.23	0.6157
2	11	9.17	12	19.35	
3	3	2.75	2	3.23	
Dysphagia					
Grade 1	1	0.92	1	1.61	0.6571
2	1	0.92	3	4.84	
3	2	1.83	0	0	
Carotid artery stenosis/occlusion/stroke	4	3.67	3	4.84	
Skull base osteoradionecrosis	1	0.92	1	1.61	

## Data Availability

The authors confirm that the data that support the findings of this study are available from the corresponding author upon request. The data are not publicly available due to privacy or ethical restrictions.

## Supplementary Materials

The following supporting information can be downloaded at: https://www.mdpi.com/article/10.3390/medicina62010076/s1, Table S1. Dosimetric data between the conventional fractionated and hyperfractionated intensity-modulated radiotherapy for patients with T4 nasopharyngeal carcinoma who received induction chemotherapy.

## Abbreviations

The following abbreviations are used in this manuscript:

AE	Adverse event
CF	Conventional fractionation
CI	Confidence interval
CTCAE	National Cancer Institute Common Terminology Criteria for Adverse Events
HF	Hyperfractionation
HR	Hazard ratio
IMRT	Intensity-modulated radiotherapy
KPS	Karnofsky performance score
NPC	Nasopharyngeal carcinoma
RT	Radiotherapy
VMAT	Volumetric modulated arc therapy
2D	Two-dimensional
3D-CRT	Three-dimensional conformal radiotherapy
18F-FDG PET	18F-fluorodeoxyglucose positron emission tomography
