# Supplementary material for: Comparison of Local Control and Toxicity in T4 Nasopharyngeal Carcinoma Patients Treated with Induction Chemotherapy and Intensity-Modulated Radiotherapy: Conventional vs. Hyperfractionated Regimens
Source: Medicina (Kaunas). 2025 Dec 30;62(1):76. doi: 10.3390/medicina62010076 (PMC12843390; doi:10.3390/medicina62010076)
Supplement: Supplementary file 1 [file medicina-62-00076-s001.zip › medicina-3997160-supplementary.pdf]

Supplement Table S1. Dosimetric data between the conventional fractionated and hyperfractionated intensity-modulated radiotherapy for patients with T4 nasopharyngeal carcinoma who received induction chemotherapy.

	Conventional fractionation (n = 109)			Hyperfractionation (n = 62)		
	mean	SD		mean	SD	p-value
Homogeneity Index	0.10	0.03		0.12	0.12	0.2252
Conformal index	0.84	0.06		0.84	0.05	0.1915
Right inner ear Dmean	35.25	9.99		41.38	11.93	0.0010
Left inner ear Dmean	35.57	10.66		41.49	11.20	0.0002
Right parotid gland Dmean	36.37	6.35		40.35	6.62	<0.0001
Left parotid gland Dmean	35.68	5.70		39.92	6.76	<0.0001
Spinal cord Dmax	38.50	5.63		41.02	7.37	0.0196
Spinal cord Dmean	23.06	6.01		26.71	6.63	0.0008
Brainstem Dmean	30.04	7.62		37.05	7.92	<0.0001
Brainstem Dmax	50.98	9.31		60.85	10.39	<0.0001
Oral cavity Dmean	34.77	5.88		37.57	5.91	0.0032
Optic chiasm Dmax	38.18	17.71		57.29	16.06	<0.0001
